# Hypohydration and Human Performance: Impact of Environment and Physiological Mechanisms

**DOI:** 10.1007/s40279-015-0395-7

**Published:** 2015-11-09

**Authors:** Michael N. Sawka, Samuel N. Cheuvront, Robert W. Kenefick

**Affiliations:** School of Applied Physiology, Georgia Institute of Technology, 555 14th Street, Atlanta, GA 30332 USA; Thermal and Mountain Medicine Division, US Army Research Institute of Environmental Medicine, Natick, MA USA

## Abstract

Body water losses of >2 % of body mass are defined as hypohydration and can occur from sweat loss and/or diuresis from both cold and altitude exposure. Hypohydration elicits intracellular and extracellular water loss proportionate to water and solute deficits. Iso-osmotic hypovolemia (from cold and high-altitude exposure) results in greater plasma loss for a given water deficit than hypertonic hypovolemia from sweat loss. Hypohydration does not impair submaximal intensity aerobic performance in cold–cool environments, sometimes impairs aerobic performance in temperate environments, and usually impairs aerobic performance in warm–hot environments. Hypohydration begins to impair aerobic performance when skin temperatures exceed 27 °C, and with each additional 1 °C elevation in skin temperature there is a further 1.5 % impairment. Hypohydration has an additive effect on impairing aerobic performance in warm–hot high-altitude environments. A commonality of absolute hypovolemia (from plasma volume loss) combined with relative hypovolemia (from tissue vasodilation) is present when aerobic performance is impaired. The decrement in aerobic exercise performance due to hypohydration is likely due to multiple physiological mechanisms, including cardiovascular strain acting as the ‘lynchpin’, elevated tissue temperatures, and metabolic changes which are all integrated through the CNS to reduce motor drive to skeletal muscles.

## Key Points

**Table:** 

Athletes performing exercise in warm-hot conditions have high sweat rates and ad libitum fluid consumption is often not sufficient to fully replace sweat losses (“voluntary dehydration”) and results in cumulative body water deficits.
A body water deficit of >2 % of body mass (~3 % of total body water for the average athlete) is defined as hypo hydration.
Hypohydration does not alter aerobic exercise performance in cold-cool conditions, sometimes impairs aerobic exercise performance in temperate conditions, and usually impairs aerobic exercise performance in warm-hot conditions.
When skin temperature exceeds 27 °C (81 °F), hypohydration impairs aerobic performance by an additional ~1 % for every 1 °C (1.8 °F) skin temperature elevation.

## Introduction

Body water and electrolyte balance perturbations are common when performing strenuous physical work and especially during exposure to the environmental extremes of heat [1], cold [2], and high altitude [3]. The resultant fluid and electrolyte losses often modify physiological strain to a particular exercise/environmental stress and sometimes impair environmental tolerance and aerobic exercise performance. Likewise, physiological modifications in fluid and electrolyte balances are consistently noted as normal physiological adaptations to these environmental extremes [4]. No single review has examined the importance of body water deficits on environmental tolerance and aerobic exercise performance during exposure to heat, cold, and high-altitude environments.

This paper provides a brief review of how body water deficits modify physiological function, sometimes environmental tolerance, and aerobic exercise performance during exposure to heat, cold, and high-altitude terrestrial environments. Prior reviews of body water deficits can be consulted that focused on body fluid balance [5], thermoregulation [6], hydration assessment [7], and exercise performance [7].

## Fluid Balance and Body Water

Water (total body water) is the principal chemical constituent of the human body. For an average young adult male, total body water represents 50–70 % of body weight [8]. Variability in total body water is primarily due to differences in body composition. Lean body mass is ~73 % water and fat body mass is ~10 % water [9, 10]. Differences in total body water attributed to age, sex, and aerobic fitness are mostly accounted for by body composition.

Total body water is distributed into intracellular fluid (ICF) and extracellular fluid (ECF) compartments. The ICF and ECF contain ~65 % and ~35 % of total body water, respectively. The ECF is further divided into the interstitial and plasma spaces. An average 70-kg male has ~42 L of total body water, therefore ICF contains ~28 L of water, whereas the ECF contains ~14 L of water with ~3.2 L in plasma and ~10.8 L in interstitium. These are not static volumes, but represent the net effects of dynamic exchange [5].

**Fig. 1:**
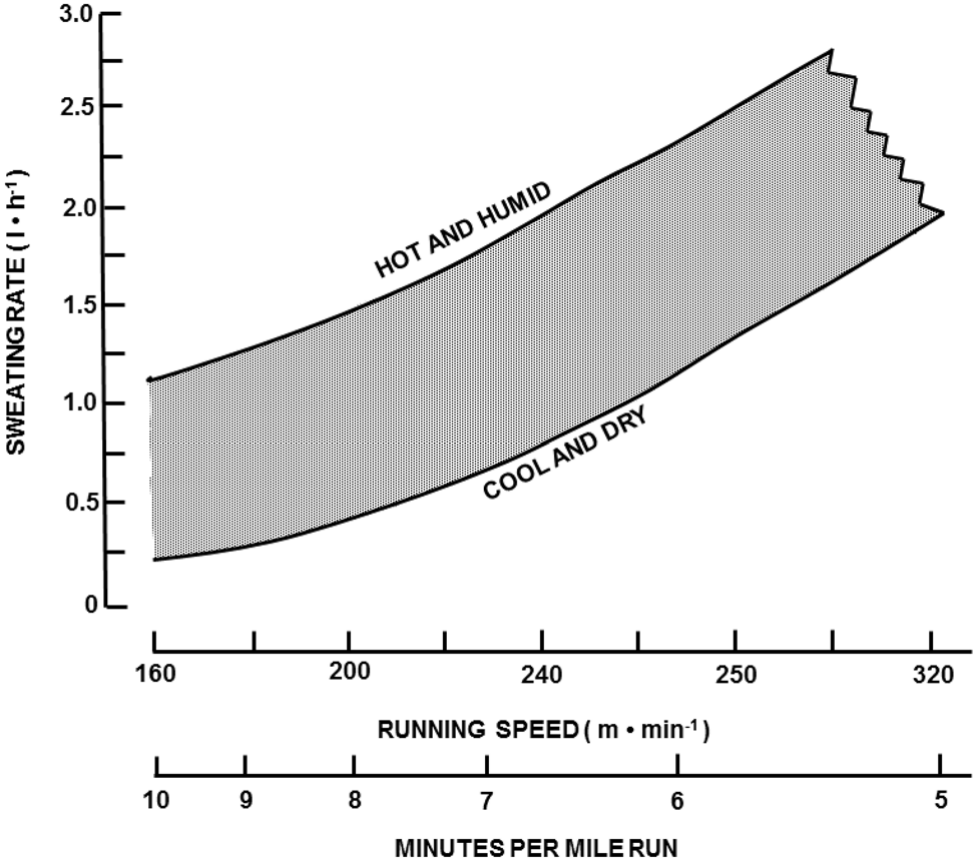
Approximation of hourly sweating rates (L/h) for runners at different running paces (m/min or min per mile) and environmental conditions (hot and humid, cool and dry). Redrawn with permission from Sawka [6]

Approximately 5–10 % of total body water is turned over daily [11], distributed via obligatory (non-exercise) fluid loss avenues. Table 1 provides the sources of daily water losses and production for sedentary and active populations [12]. Respiratory water losses are influenced by the temperature and humidity of inspired air and the pulmonary ventilatory volume. Metabolic water is formed by oxidation of substrates and is roughly offset by respiratory water losses. Urine output generally approximates 1–2 L per day but can be increased by an order of magnitude when consuming large volumes of fluid. This large capacity to vary urine output represents the primary avenue to regulate net body water balance across a broad range of fluid intake volumes and losses from other avenues [13]. Sweat losses vary widely and depend upon the physical activity level and environmental conditions: with ambient temperature, radiant heat load, high humidity, and elevated metabolic rate all markedly elevating sweating requirements [14, 15]. Figure 1 provides an approximation of hourly sweating rates for athletes running at different speeds and exposed to different heat stress conditions [6]. Sweating rates of >1.0 L per hour are common due to either high metabolic rates and/or environmental heat stress.

**Table 1:** Daily water losses and production

Source	Loss (mL/day)	Production (mL/day)
Respiratory loss	−250 to –350	
Urinary loss	−500 to –1000	
Fecal loss	−100 to –200	
Insensible loss	−450 to –1900	
Metabolic production		+250 to +350^a^
Total	−1300 to −3450	+250 to +350
Net loss (sedentary)	−1050 to −3100	
Sweat losses in various sports	−455 to −3630	
Net loss (athlete)	−1550 to −6730	

Adapted from Sawka et al. [12], with permission

^a^Metabolic water production based on 2500–3000 kcal daily energy expenditure. Additional water production with exercise is assumed offset by parallel respiratory losses

Net body water balance (loss = gain) is regulated remarkably well day-to-day as a result of thirst and hunger drives, coupled with ad libitum access to food and beverages to off-set water losses [7]. This is accomplished by an intricate interplay between neuroendocrine and renal responses to body water volume and tonicity changes [16], as well as non-regulatory social–behavioral factors [17]. These homeostatic responses collectively ensure that small degrees of over- and under-hydration are readily compensated for in the short term [13].

**Fig. 2:**
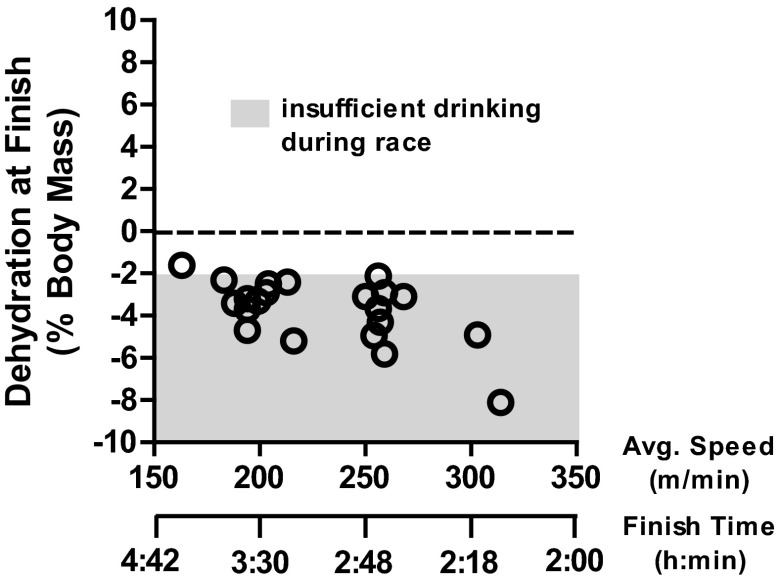
Plot of average running speed and finish time for 42 km versus the magnitude of post-race hypohydration (dehydration, % body mass loss) level at finish when drinking ad libitum. Redrawn with permission from Cheuvront et al. [25]

There is excellent support that, over many hours, if adequate fluid and food are available, these homeostatic responses allow humans to sustain euhydration [13, 18]. For example, an Institute of Medicine analysis of NHANES II data on the first to tenth deciles of individuals consuming fluid volumes demonstrated that serum osmolality values were similar and that all individuals were likely euhydrated [13]. However, during periods of high sweating rates, such as during strenuous physical exercise in hot weather, humans practicing ad libitum drinking can markedly under-consume fluids [18–22] and thus incur body water deficits [23, 24]. Figure 2 plots body water deficits incurred by marathon runners practicing ad libitum drinking at different paces, even in mild conditions ranging from cold to warm [25]. Note that most runners achieved body water deficits >2 % of body mass.

## Body Water Deficits

Hypohydration is defined as a body water deficit greater than normal daily fluctuation [7]. Changes in hydration status can be assessed by a variety of body measures [26]; however, they all have specific limitations [7, 27]. Because of low measurement variability, changes in body mass provide the most sensitive and simplest measure to determine acute changes in body water for all types of dehydration [13, 16, 26]. Of course, body mass measures are dependent upon subjects remaining in energy balance and accurate book keeping of intake (e.g., food, fluid) and output (e.g., urine, feces). Body water deficits >2 % of body mass exceed 2 standard deviations in normal body mass variability [18, 28] and represent an approximate threshold, based on plasma volume reductions and plasma osmolality increases, where compensatory fluid regulatory actions occur [16]. Therefore, we define hypohydration as >2 % of body mass loss (~3 % of total body water) from water deficits [7, 24] as this has >95 % chance of exceeding normal daily fluctuations in body water.

Incomplete fluid replacement decreases total body water, and as a consequence of free fluid exchange, affects each fluid space [29, 30]. For example, Nose and colleagues [31] determined the distribution of body water loss among the fluid spaces as well as among different body organs during hypohydration. They thermally dehydrated rats by 10 % of body weight, and after the animals regained their normal core temperature, the body water measurements were obtained. The fluid deficit was apportioned between the ICF (41 %) and ECF (59 %) spaces. Regarding organ fluid loss, 40 % came from muscle, 30 % from skin, 14 % from viscera and 14 % from bone. Neither the brain nor liver lost significant water content as measured from wet and desiccated organ weights. They concluded that hypohydration results in water redistribution largely from the ICF and ECF spaces of muscle, gut and skin in order to defend blood volume.

Although earlier research measuring wet/dry weight of excised tissues suggested that with severe hypohydration (10 % of total body water) brain water content was preserved [31], recent studies employing functional magnetic resonance imaging (fMRI) suggest acute brain anatomical alterations with hypohydration consistent with fluid loss [32–34]. Brain ventricle volume has been demonstrated to expand with hypohydration [32, 33, 35], which would be consistent with fluid loss from surrounding brain tissues. Streitburger et al. [34] examined the impact of dehydration (~2 % of body mass incurred over 3 days) on brain gray matter, white matter, and cerebral spinal fluid by fMRI. They reported that dehydration decreased both gray matter and white matter volume in the temporal and sub-gyral parietal areas and left inferior orbito-frontal region and the extra-nuclear region. In addition, they corroborated that dehydration causes expansion of the ventricle system (lateral, third, fourth). These changes in brain structure gray matter remained fairly constant over the 3 days of chronic progressive dehydration. Therefore, hypohydration mediated changes in brain structure and function may alter the integration of afferent information during rest and exercise.

**Fig. 3:**
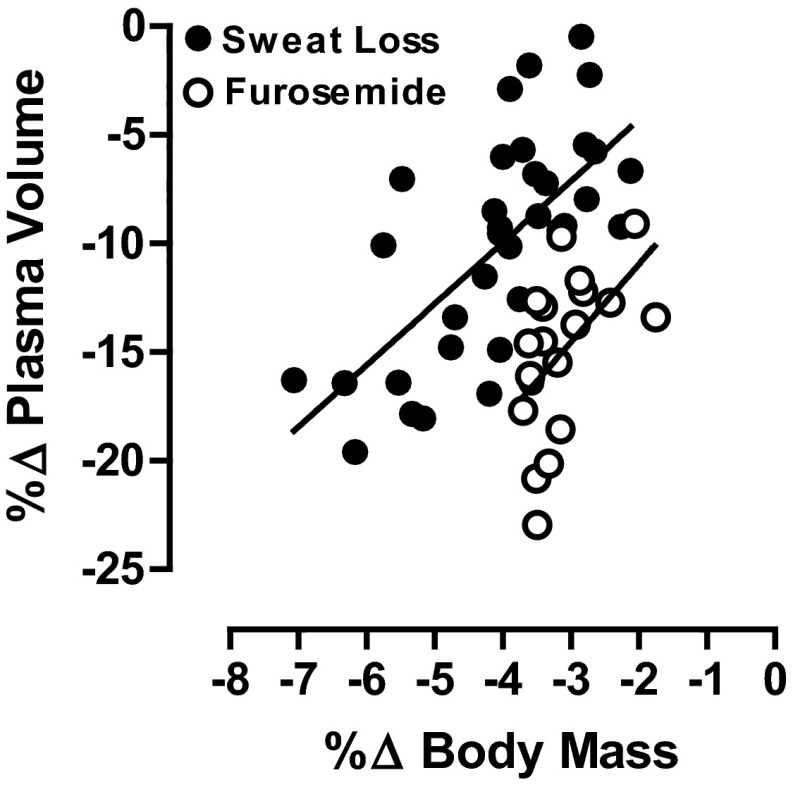
Linear regression of plasma volume change (%∆ plasma volume) and hypohydration level (%∆ body mass) after induction of hypotonic (sweat) and isotonic (diuretic) body water losses. Redrawn with permission from Cheuvront et al. [37]

Sweat-induced hypohydration will decrease plasma volume and increase plasma osmotic pressure in proportion to the decrease in total body water [13, 36]. The reduction in plasma (blood) volume with the same vascular space size is often referred to as an absolute hypovolemia. Plasma volume decreases because it provides the fluid for sweat, and osmolality increases because sweat is hypotonic relative to plasma. Sodium is the primary ion responsible for the elevated plasma osmolality. The plasma hyperosmolality acts to mobilize fluid from the intracellular to the extracellular space to enable plasma volume defense in hypohydrated subjects [5]. Diuretics (e.g., furosemide) can be used to model the type of water and solute loss observed in cold and high terrestrial altitude environments [7]. Diuretic-induced hypohydration generally results in an iso-osmotic hypovolemia, with a much greater ratio of plasma loss to body water loss than either exercise or heat-induced hypohydration. Relatively less intracellular fluid is lost after diuretic administration, since there is no extracellular solute excess to allow osmotic redistribution of water from the intracellular space [16]. Figure 3 provides plasma volume reductions with hypohydration (percent change in body mass) after sweat-induced (hypertonic) and diuretic-induced (isotonic) body water deficits [37]. Consistent with this, the environmental stressors of cold [38] and high altitude [3] stimulate diuresis with solute losses, thus inducing an isotonic hypovolemia.

## Environmental Temperatures and Exercise Performance

**Fig. 4:**
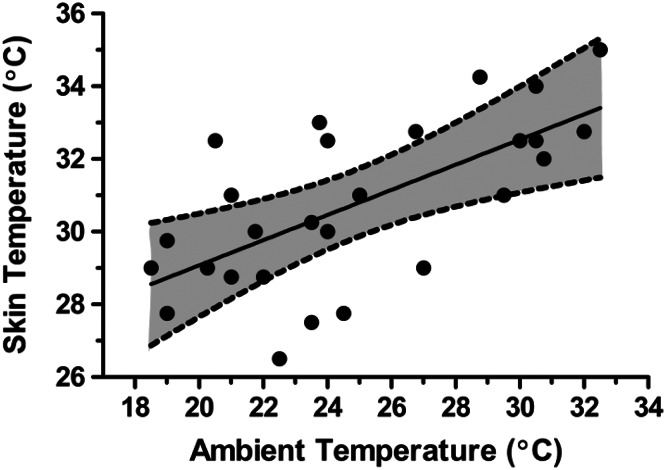
Relationship (regression line) between ambient (shaded dry bulb) temperature (°C) and skin temperature (°C) during aerobic exercise while wearing minimal clothing. The *broken lines* and *grey shading* represent 95 % confidence intervals. Figure was drawn from data in Adams [42]

During exercise in the heat, the most significant physiological burden is to support high skin blood flow for heat dissipation [39, 40]. Skin temperature (*T*_sk_) is elevated in proportion to ambient temperature and humidity [41], while core temperature (*T*_c_) is elevated in proportion to exercise intensity and is largely independent of the environment during compensable heat stress [1, 39]. Warm–hot skin is associated with a greater skin blood flow and cutaneous venous compliance, which augments cardiovascular strain [1, 39]. The increase in vascular fluid volume (such as from cutaneous vasodilation and compliance) with no change in plasma (blood) volume is often referred to as relative hypovolemia. Figure 4 illustrates the approximate relationship between ambient temperature conditions and skin temperature during aerobic exercise while wearing minimal clothing [42]. For this review, we define cool/cold skin as <30 °C, warm skin as 30–34.9 °C and hot skin as 35 °C and above. We recognize that skin temperature effects are a continuum and the *T*_sk_ to *T*_c_ gradient alters these relationships.

Table 2 illustrates the effects of different *T*_sk_ and *T*_c_ combinations on estimated whole body skin blood flow requirements calculated from the equation of Rowell [43] applied to exercise-heat stress. An elevated *T*_sk_ increases skin blood flow at any given *T*_c_, while an elevated *T*_c_ reduces skin blood flow requirements at any given *T*_sk_. An often under-appreciated point is at any given skin temperature, an elevation in core temperature reduces whole body skin blood flow and can be viewed as a positive response for sustaining aerobic performance in the heat. Generally, warmer skin is associated with greater skin blood flow responses and greater heart rate elevations during exercise in the heat [39, 40]. The result is a reduction in cardiac filling and a challenge to sustain blood pressure [44].

**Table 2:** Estimated whole body skin blood flow (SkBF) requirements^a^ during prolonged, severe running exercise^b^ at different body core (*T*_c_) and skin (*T*_sk_) temperatures

*T*_c_ (°C)	*T*_sk_ (°C)	Gradient (°C)	SkBF (L/min)
38	30	8	1.1
38	34	4	2.2
38	36	2	4.4
39	36	3	2.9

Adapted from Kenefick et al. [66], with permission

^a^Equation for skin blood flow: *Q*_s_ = 1/*C* × *h*/(*T*_c_−*T*_sk_), where, *C* specific heat of blood ≈0.87 kcal/°C/L, *h* heat production (kcal/min), *Q*_s_ skin blood flow [43]

^b^Net heat production (7.7 kcal/min) estimated using 60 kg body mass and 325 m/min running velocity (approximate pace for men’s world class 42 km footrace) after subtracting for work (20 % efficiency) and 50 % dry and evaporative heat losses

It is important to recognize that when skin temperature is elevated there is an increased requirement on sweat secretion and evaporation to regulate body temperature. Thus, during exercise in the heat with high sweat rates, there is the simultaneous problem of reduced plasma volume from hypohydration while skin blood flow requirements are elevated. It will be discussed later (Sect. 6: Mechanisms of Impaired Aerobic Performance) that the dual perturbation of reduced plasma volume (absolute hypovolemia) with increased skin blood flow (relative hypovolemia) is likely an important physiological prerequisite to impair aerobic performance.

It is generally accepted that heat stress alone will impair aerobic performance [40], and that cold stress alone does not impair aerobic performance unless the temperature is sufficient to adversely impact on skeletal muscle function and nerve conduction [45]. The earliest scientific experiments regarding body water deficits and exercise capacity were conducted by the military and clearly concluded that in hot environments fluid replacement better sustained marching and military endurance performance in laboratory and field trials [19, 20, 46]. Subsequently, submaximal intensity and maximal intensity aerobic performance tests were developed and widely employed. Physiological reviews examining the impact of water deficits on submaximal and maximal aerobic performance again concluded that hypohydration impaired aerobic performance in warm and hot environments [6, 7, 13, 47, 48]. Despite these consistent findings, there has recently been some controversy within the sporting community regarding these points [49].

**Fig. 5:**
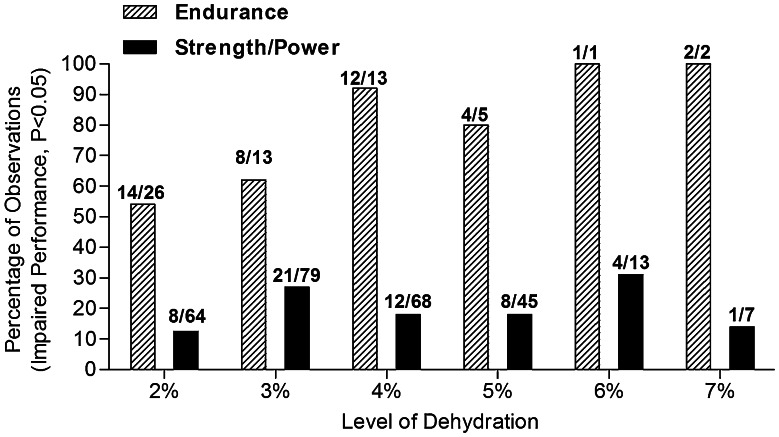
Summary of a literature review of hypohydration level (percent change in body mass) effects on endurance (34 studies) and power (43 studies) performance. The *y* axis is the percentage of observations that demonstrated impaired performance (*P* < 0.05) with the appropriate fraction above each data *bar*. Redrawn with permission from Cheuvront and Kenefick [7]

Figure 5 provides the summary of a literature review on hypohydration level effects on endurance (34 studies) and strength/power (43 studies) performance [7]. Consistent with what had been concluded by others [13, 24], body water deficits usually did not appear to significantly impair strength/power tasks. In contrast, endurance performance was significantly impaired in the vast majority of studies when body water deficits exceeded 3 % of body mass. However, that particular literature analysis did not factor in the impact of environmental heat stress [7].

Table 3 provides a review of studies examining the impact of hypohydration (≥2 % body mass) on submaximal aerobic performance, ordered by the ambient temperature in which the exercise protocols were conducted (cold on top and uncompensable heat stress on the bottom). It is assumed that this approximates the severity of heat strain; however, since evaporative requirements and maximal environmental evaporative capacities were not reported, this order might not be precise. Submaximal aerobic performance was evaluated by either time-to-exhaustion (TTE) or time-trial (TT) protocols, and since the TTE protocols are generally longer in duration they might be expected to demonstrate larger percentage changes than the TT protocols [40]. During cold stress (2 and 10 °C) environments, hypohydration did not (0 of 2 studies) alter aerobic performance. During temperate conditions (20–24 °C), hypohydration sometimes (4 of 7 studies) impaired aerobic performance. During warm–hot conditions (>25 °C to uncompensable heat stress), hypohydration usually (8 of 9 studies) impaired aerobic performance.

**Table 3:** Review of literature on impact of ambient temperature on hypohydration effects on submaximal aerobic performance

Study	*N*	Test	Environment (°C)	BML (%)	Reduction (%)
Cheuvront et al. [52]	8	TT	2	3	ND
Kenefick et al. [50]	8	TT	10	4	ND
Fallowfield et al. [67]	8	TTE	20	2	−24
Oliver et al. [68]	13	TT	20	3	ND
Cheuvront et al. [52]	8	TT	20	3	−8
Kenefick et al. [50]	8	TT	20	4	ND
McConell et al. [69]	8	TT	21	2	ND
McConell et al. [70]	7	TTE	21	3	−47
Merry et al. [71]	12	TT	24	2	−9
Castellani et al. [51]	7	TT	27	4	−17
Ebert et al. [72]	8	TT	29	2	−29
Kenefick et al. [50]	8	TT	30	4	−12
Below et. al. [73]	8	TT	31	2	−6
Walsh et al. [74]	6	TTE	32	2	−31
Cheung et al. [76]	11	TT	35	3	ND
Kenefick et al. [50]	8	TT	40	4	−23
Cheung and McLellan [75]	15	TTE	UCHS	2	−14
Sawka et al. [77]	17	TTE	UCHS	5	−54

*BML* body mass loss, *ND* no determination, *TT* time-trial performance test, *TTE* time to exhaustion performance test, *UCHS* uncompensable heat stress

**Fig. 6:**
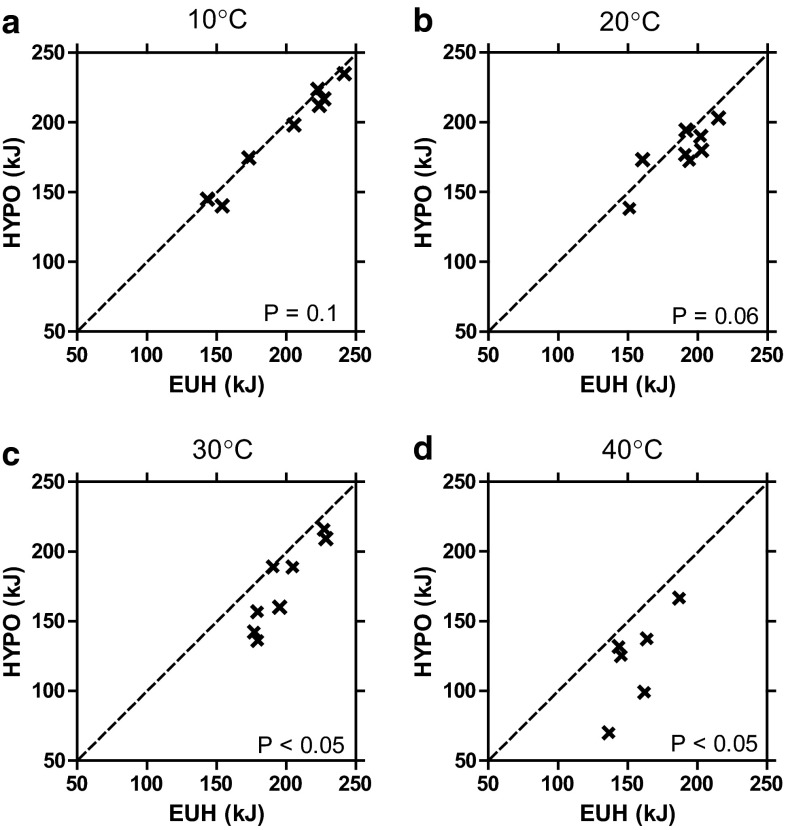
Individual data for time-trial performance (kJ) when euhydrated (*EUH*) and hypohydrated (*HYPO*, 4 % body mass loss) in **a** 10 °C, **b** 20 °C, **c** 30 °C and **d** 40 °C environments. Adapted with permission from Kenefick et al. [50]

Figure 6 provides individual subject data for the only study to examine the impact of submaximal aerobic performance at a variety of environmental conditions using similar protocols [50]. Note in the 10 and 20 °C environments, aerobic performance was not different when euhydrated and hypohydrated; however, in the warm (30 °C) and hot (40 °C) environments, aerobic performance was impaired. It can be noted that as ambient temperature increased there was almost always an impaired aerobic performance. Therefore, although occasional individual exceptions might be found, almost all subjects demonstrated hypohydration-mediated impaired aerobic performance in warm and hot environments.

**Fig. 7:**
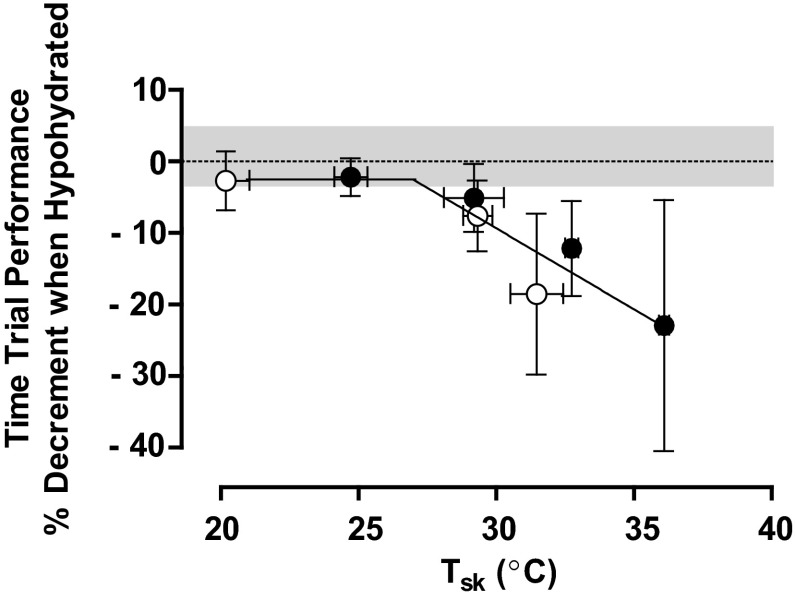
Percentage impairment in submaximal intensity aerobic performance (time-trial) from euhydration as a function of skin temperature (*T*_sk_) when hypohydrated by 3–4 % of body mass. *Regression line* indicates that at a *T*_sk_ intercept of ~27 °C, the percentage decrement in aerobic exercise performance declines linearly by ~1.3 % for each 1 °C rise in *T*_sk_. The best-fit equation for the second linear line segment is *y* = −1.26*x* + 26.37. Data are means [*error bars* are 95 % confidence interval] for paired observations from three studies [50–52]. *Filled circles* represent 15-min time-trial tests, *open circles* represent 30-min time-trial tests. *Gray area* represents the collective percent coefficient of variation of repeated practice time-trials (when euhydrated) in temperate conditions (~3.5 %). Adapted with permission from Sawka et al. [53]

Figure 7 plots the impact of hypohydration on submaximal aerobic performance from several hypohydration studies [50–52] conducted in our laboratory [53]. These studies employed similar procedures over a broad range of *T*_sk_ from 20 to 36 °C. Performance was plotted as a function of *T*_sk_, as modifiers of environmental evaporative capacity will have a direct effect on this parameter. Segmented regression was used to approximate the statistical *T*_sk_ threshold for performance impairment using individual study data points (*n* = 53 paired observations). The threshold which best minimized the residual sums of squares was shown as 27.3 °C and warmer skin accentuated the performance impairment by ~1.5 % for each additional 1 °C rise in *T*_sk_. Therefore, as ambient conditions become warmer and elevate cutaneous vasodilation, the adverse impact of hypohydration is clearly demonstrated [53].

In addition to impaired submaximal intensity aerobic performance, hypohydration has been reported to consistently impair maximal intensity aerobic performance. Several previous review papers have addressed the maximal intensity aerobic performance impairment and can be consulted [7, 47].

## Terrestrial High Altitude

Physical exertion at high altitude likely induces sweat rates comparable to those at sea level for a given heat strain [15], and respiratory water loss is elevated at high altitude [3]. Furthermore, with high-altitude exposure there is a proportionate reduction in plasma volume that is due to both diuresis and reduced plasma proteins [54]. Therefore, at high altitude, hypohydration can occur from both sweat loss and adaptations to that environment. In addition, acute high-altitude exposure induces cutaneous and skeletal muscle vasodilation during exercise, thus possibly inducing relative hypovolemia [55].

**Fig. 8:**
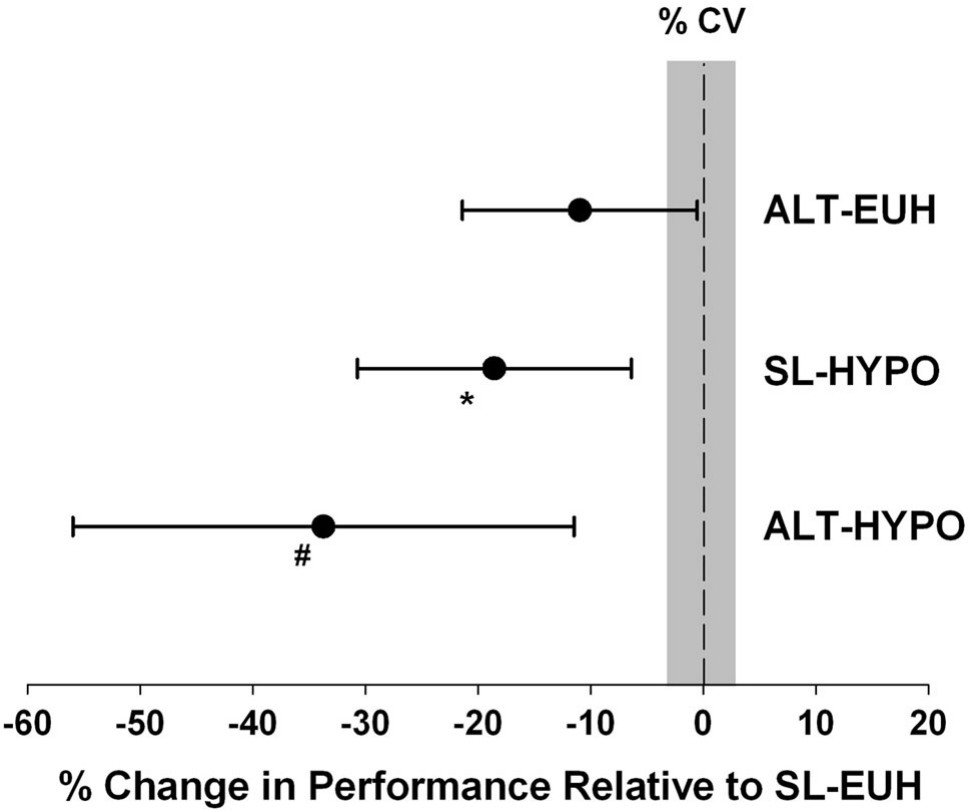
Percent change in aerobic performance (time-trial) from euhydrated sea-level (*SL-EUH*) [zero reference] when euhydrated at high altitude (*ALT-EUH*), hypohydrated at sea level (*SL-HYPO*) or hypohydrated at high altitude (*ALT-HYPO*). Environmental temperature is 27 °C, high altitude is 3048 m, hypohydration is 4 % body mass loss and coefficient of variation (CV) is 3.1 %. *Gray area* represents collective percent coefficient of variation of repeated practice time-trials (when euhydrated). *P = 0.04, ^#^ *P* < 0.001. Data are mean and the *error bars* represent the 95 % confidence intervals. Adapted with permission from Castellani et al. [51]

Castellani and colleagues [51] examined the impact of hypohydration (4 % body mass) on aerobic performance. Their subjects performed time-trial tests in a warm environment (27 °C) when euhydrated and hypohydrated at both sea level and simulated high altitude (3048 m). Figure 8 provides the percent change in aerobic performance from euhydration sea level to euhydration high-altitude, sea-level hypohydration and high-altitude hypohydration trials. They found that compared with aerobic performance at sea level when euhydrated, performance was impaired by −17 % with sea-level hypohydration, −11 % when euhydrated at high altitude and −34 % when hypohydrated at high altitude. Therefore, altitude and hypohydration had additive effects on impairing performance.

## Mechanisms of Impaired Aerobic Performance

Hypohydration impairs aerobic performance when heat stress is present and this adverse impact is accentuated with high-altitude exposure. Heat stress is unique because it induces considerable cardiovascular strain to support skin blood flow requirements to the induced hyperthermia (elevated skin and usually core temperatures). Nybo and colleagues [40] recently provided an extensive review of physiological mechanisms impairing aerobic performance in warm–hot environments and proposed an integrated model. Those authors concluded that “One simple explanation for the underlying physiological mechanisms will not suffice, because a variety of factors change in parallel and some factors may affect performance independently or they may interact with other hyperthermia or exercise-induced factors”. Table 4 briefly summarizes the physiological mechanisms impairing aerobic performance during heat stress as summarized by Nybo and colleagues [40]. It is critical to note that hypohydration exacerbates all of the proposed physiological mechanism(s) thought to limit aerobic performance from just heat stress alone.

**Table 4:** Physiological mechanisms potentially contributing to impaired exercise performance in warm–hot environments

System	Examples of mechanisms
Cardiovascular	Blood pressure, blood flow to brain and skeletal muscles, oxygen delivery and metabolite removal
Central nervous system and neurobiological	Cerebral metabolism, neurotransmitter levels, temperature
Peripheral muscular factors	Temperature, metabolic, afferent feedback
Psychological	Thermal comfort, rating of perceived exertion, motivation and expectations
Respiration	Hypocapnia, alkalosis, breathing sensations

Adapted from Nybo et al. [40]

Several examples of how hypohydration exacerbated the physiological mechanism(s) associated with impaired aerobic performance in the heat are provided. It is well recognized that hypohydration will further increase cardiovascular strain and make it more difficult to sustain the required cardiac output during aerobic exercise [56, 57]; hypohydration can impair skeletal muscle blood flow [58] while heat stress alone does not. Hypohydration restricts cerebral blood flow (but likely not cerebral oxygen uptake) during high-intensity exercise in the heat [59]. Likewise, hypohydration elevates core temperature [36], skeletal muscle glycogen usage [60], fatigue/discomfort [61], respiratory alkalosis [62], afferent feedback [63], skeletal muscle motor-unit recruitment [64], and brain function [33]. Clearly, a multitude of physiological mechanism(s) contributing to impaired exercise-heat performance [40] are further aggravated by hypohydration [7], thus providing mechanism(s) for the hypohydration-impaired aerobic performance during heat stress.

Simultaneous absolute hypovolemia and relative hypovolemia appears to be a prerequisite for hypohydration to impair aerobic performance. Both types of hypovolemia elevate cardiovascular strain and thus we believe that challenges to blood pressure regulation often may be a ‘lynchpin’ mechanism in impairing aerobic performance with hypohydration [19, 39, 40]. It should be noted that the frequent ‘sporting literature’ explanation of hyperthermia-induced fatigue of a ‘critical core temperature’ is poorly supported by physiological studies [37, 40, 53]. As previously discussed (Sect. 4, paragraph 2), a moderately elevated core temperature as induced by hypohydration [13, 19, 36] can likely often be beneficial in reducing skin blood flow requirements, thus helping to minimize cardiovascular strain and sustain aerobic performance [39, 45, 53]. In addition, thirst might contribute to impaired performance.

Table 4 does not include a recent study [65] that purports that hypohydration with warm skin does not impair aerobic exercise performance. Wall and colleagues [65] employed intravenous infusion to partially rehydrate subjects, and showed that despite a 3 % body mass loss, time-trial performance was not altered. Importantly, their subjects’ heart rate responses were not elevated by hypohydration while performing exercise at the same intensity as when euhydrated. Studies employing hypohydration with exercise at a given intensity will consistently demonstrated elevated heart rates. This strongly suggests that the saline infusions likely restored plasma volume and cardiac preload (thus filling) to negate the adverse cardiovascular impact of hypohydration (i.e., absolute hypovolemia).

## Conclusions

Hypohydration accentuates the aerobic performance impairments observed in hot and high-altitude environments. Impaired aerobic performance when hypohydrated during heat stress has been consistently reported for decades for both laboratory and field trials, and numerous physiological mechanisms have been identified to explain such impairments. All of the physiological mechanisms believed to impair aerobic performance with heat stress alone are markedly aggravated further with hypohydration. It is possible that simultaneous absolute and relative hypovolemia is a prerequisite for performance impairments in warm–hot and high-altitude environments and that the cardiovascular system often is the important ‘lynchpin’ for impairing aerobic performance when hypohydrated.
